# Supplementary material for: Modelling the epidemiology of residual Plasmodium vivax malaria in a heterogeneous host population: A case study in the Amazon Basin
Source: PLoS Comput Biol. 2020 Mar 13;16(3):e1007377. doi: 10.1371/journal.pcbi.1007377 (PMC7108741; doi:10.1371/journal.pcbi.1007377)
Supplement: S1 File — (PDF) [file pcbi.1007377.s001.pdf]

Parameter estimation process

The SIS compartmental model does not consider a latent period. We therefore assume that individuals recover and become susceptible again in an average of 28 days after infection (equivalently, with a recovery rate $\gamma = 1/28$ per day). This time interval corresponds approximately to the duration of the post-treatment prophylactic effect of a full course of chloroquine (CQ; total dose, 25 mg of base/kg over 3 days) and primaquine (PQ; 0.5 mg of base/kg/day for 7 days), the antimalarial drugs used for radical cure of vivax malaria in Brazil [1]. Remaining parameters were estimated by simultaneously fitting two sets of empirical data: (a) the age-specific malaria incidence density in the urban population of Mâncio Lima $(D_1 = \{(k, \tilde{y}_{1k})\}_{k=0}^{80})$ and (b) the number of vivax malaria episodes notified per urban resident over 12 months of follow-up $(D_2 = \{(k, \tilde{y}_{2k})\}_{k=0}^{6})$. This approach contrasts with previous attempts to fit similar SIS models to age-related malaria prevalence or incidence data in that we also consider the overall frequency distribution of malaria episodes in the population [2, 3]. First, assuming equilibrium conditions, the system of ODEs was simulated, in age domain, from age 0 to 80 in order to generate incidence profiles over age and risk group. Next, we reprofiled incidence over age according to the population age structure determined by our census survey and computed a distribution of the number of cases experienced per person over 12 months. Parameter estimation was performed with the software Matlab, using PESTO (Parameter EStimation Toolbox; [4]). We assume that the residuals between model outputs and data are normally distributed, with unknown standard deviations. Our optimisation process maximized the likelihood (Equation S1) of observing both datasets, that is,

$$L(D_1, D_2, \theta) = \prod_{k=0}^{80} \frac{1}{\sigma_1\sqrt{2\pi}} \exp\left(-\frac{\left(\tilde{y}_{1k} - y_1(k)\right)^2}{2\sigma_1^2}\right) \cdot \prod_{k=0}^{6} \frac{1}{\sigma_2\sqrt{2\pi}} \exp\left(-\frac{\left(\tilde{y}_{2k} - y_2(k)\right)^2}{2\sigma_2^2}\right), \tag{S1}$$

in which y_1 is the model output for age-specific malaria incidence densities, y_2 is the model output for the number of cases per person over 12 months, σ_1 is the standard deviation of the measurement noise for y_1, and σ_2 is the standard deviation of the measurement noise for y_2. We optimized the model fitting considering that the HR group comprised 10%, 15%, 20%, 25% or 30% of the hosts; although where exactly we partition what is conceivably a continuous risk distribution is somewhat arbitrary we informed this selection on likelihood values. To ensure that the observed maximum is global, we performed 30 multi-starts initialised with randomly sampled parameter values following a Latin hypercube. We also used PESTO to derive 95% credible intervals for each parameter by using Monte-Carlo Markov Chain methods considering 10^5 iterations.

References

1. Ministry of Health of Brazil. Practical guidelines for malaria therapy [in Portuguese]. Brasília, Ministry of Health of Brazil. Brasília, Brazil: Ministry of Health of Brazil; 2010. Available from: http://bvsms.saude.gov.br/bvs/publicacoes/guia_pratico_malaria.pdf.
2. Águas R, White LJ, Snow RW, Gomes MGM. Prospects for malaria eradication in sub-Saharan Africa. PLoS ONE 2008; 3: e1767.

3. White MT, Walker P, Karl S, Hetzel MW, Freeman T, Waltmann A, et al. Mathematical modeling of the impact of expanding levels of malaria control interventions on *Plasmodium vivax*. Nat Commun 2018;9:3300.

4. Stapor P, Weindl D, Ballnus B, Hug S, Loos C, Fiedler A, et al. PESTO: Parameter EStimation TOolbox. Bioinformatics 2018 15;34:705-707.
