# Supplementary material for: Modelling the epidemiology of residual Plasmodium vivax malaria in a heterogeneous host population: A case study in the Amazon Basin
Source: PLoS Comput Biol. 2020 Mar 13;16(3):e1007377. doi: 10.1371/journal.pcbi.1007377 (PMC7108741; doi:10.1371/journal.pcbi.1007377)
Supplement: Attachment — Submitted filename: Rebuttal_R1.pdf [file pcbi.1007377.s002.pdf]

PCOMPBIOL-D-19-01472

Modelling the epidemiology of residual *Plasmodium vivax* malaria in a heterogeneous host population: a case study in the Amazon Basin

Editor's comments:

Thank you very much for submitting your manuscript 'Modelling the epidemiology of residual *Plasmodium vivax* malaria in a heterogeneous host population: a case study in the Amazon Basin' for review by *PLOS Computational Biology*. Your manuscript has been fully evaluated by the *PLOS Computational Biology* editorial team and in this case also by independent peer reviewers. The reviewers appreciated the attention to an important problem, but raised some substantial concerns about the manuscript as it currently stands. While your manuscript cannot be accepted in its present form, we are willing to consider a revised version in which the issues raised by the reviewers have been adequately addressed. We cannot, of course, promise publication at that time.

> We thank the reviewers for their detailed review of our manuscript and the many insightful suggestions provided. We have carefully addressed all comments and suggestions, and changed the text accordingly.

Reviewer #1:

Thank you for inviting me to review the manuscript titled "Modelling the epidemiology of residual *Plasmodium vivax* malaria in a heterogeneous host population: a case study in the Amazon Basin" by Corder et al. The manuscript used mathematical models to estimate the risk of malaria in the Amazon region while accounting for geographical heterogeneity of the study area. I have the following comments for major revision.

Major comments:

1. At times the introduction blurred into the results, and the results blurred into the discussion. For example,

(i) Line 82-89 should be part of the results and not introduction.

(ii) Line 460-464 should be moved to the discussion.

The results section should only consist of results. May I suggest you align and insert these sentences into the first paragraph of the discussion.

We thank the reviewer for pointing out these issues. We have removed sentence (i) from the Introduction. Its content is detailed in Results where two dedicated sections titled “A 20% fraction of high-risk individuals accounts for 86% of the community-wide malaria burden” and “High-risk individuals develop immunity and constitute a clinically silent reservoir of infection”, respectively, can be found. We have moved the sentence (ii) to the Discussion section:

Last sentence of 4th paragraph: “Estimates of the proportions of asymptomatic infections that are patent (consistent with RI close to 1/2) vary by one order of magnitude, from 4.5% [37] to 46.7% [32], in Amazonian populations.”

Firth sentence of 5th paragraph: “The importance of characterising the malaria reservoirs in endemic regions has recently been highlighted [38] and these results further underscore how essential this information is to inform elimination programmes and for properly planning control interventions.”

2. The methodology (mathematical models) is too long consisting about 8 pages of different techniques. Could the authors summarize the techniques into a maximum of 2 pages and moved the detailed mathematical models to supplementary materials.

We agree that 8 pages describing the mathematical models are too much and thank to the reviewer for pointing it out. We provide a brief description of the mathematical models in the main text (3.5 pages in double spacing, including the figure legends) and the details were added to the supporting information S1 Text, as suggested by the reviewer.

Minor comments:

3. Line 52-53: Insert some references here.

Done.

4. The authors mentioned in line 52-53 that varying malaria risks have been observed in several towns and cities in Africa countries. However, the example provided was in the city of Brazzaville (Not an African city/country).

We added a couple of new references, in addition to the one cited about Brazzaville, capital and largest city of the Republic of Congo, a country located on the western coast of Southern Africa.

5. Line 54-58: Insert references here.

Done.

6. Line 77-78: It will be nice to have a sentence describing what residual transmission is.

Done (sentence and reference added).

7. Line 216: …the antimalaria drugs used for radical cure of vivax malaria in Brazil. Provide a reference for this statement.

Done. Reference added to the Supplementary Text S1, where the malaria treatment regimens currently used in Brazil are described.

8. Line 377. Here and throughout the manuscript insert "95% CI" in front of the 95% Credible Interval (CI). Eg. 0.0883 [95% CI: 0.0801-0.1189]

Thank you. This has been corrected.

9. Line 506: "…the model predicts that as much as 25 past malaria…" By 25 in this statement, are they saying 25% of past malaria?

We mean that 25 malaria episodes are experienced, on average, before the risk of clinical malaria decreases by 50%. We added some further clarifications to the main text.

Reviewer #2:

This manuscript presents fine work. It is based on very good data: a town in Brazil with high malaria incidence, monitored during one year, person by person. This is a piece of exceptional data. The work addresses the question of risk heterogeneity, an important one. And it proposes a mathematical model that is shown to fit very well the data.

So far, so good, but I would like to raise some points.

1- I don’t really get the point why the two risk classes do not interact. Are the people in those two risk classes somehow geographically separated? Because, if they are mixed, wouldn’t it be the case of a HR person being a potential spreader in the LR population. Please clarify the assumptions of the model at this point.

Interactions among individuals are not specified in our model (irrespective of whether they belong to different risk classes or to the same class). The model does not represent transmission explicitly. All individuals are exposed to a constant source of infection, termed as the force of infection (lambda). Different risk classes are then introduced to differentiate individuals according to how easily they acquire infection from that common source. To prevent misleading our readers we removed the statement “individuals do not move between LR and HR groups” (previously in Materials and Methods) and refrain from referring to the model as a “transmission model” (instead we use the term “mathematical model” where we see potential for confusion).

2-Could you make explicit the parameters that are being fitted. I understand that including age classes is realistic, but also introduces new parameters. Expression (1), for instance has two parameters. Lambda zero is not a problem, but the parameter “k”in Eq.(1) seems arbitrary. Is it a free parameter to be fitted?

Parameter “k” determines how steeply the force of infection increases in early ages and “c” controls the value at birth. Fixing parameters “c” and “λ_0”, the figure below shows the variation in $\lambda(a)$ by varying only parameter “k”.

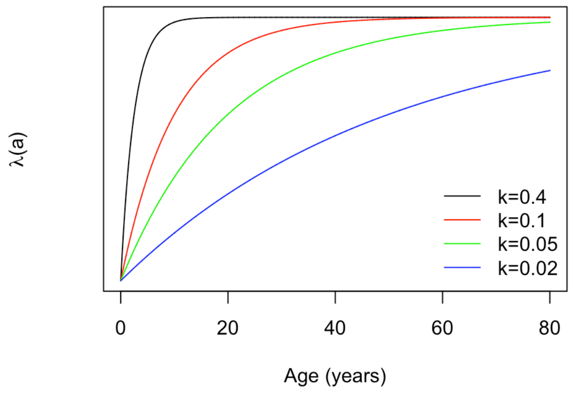

Now, fixing parameters "k" and "λ_0", the figure below shows the variation in $\lambda(a)$ by varying only parameter "c".

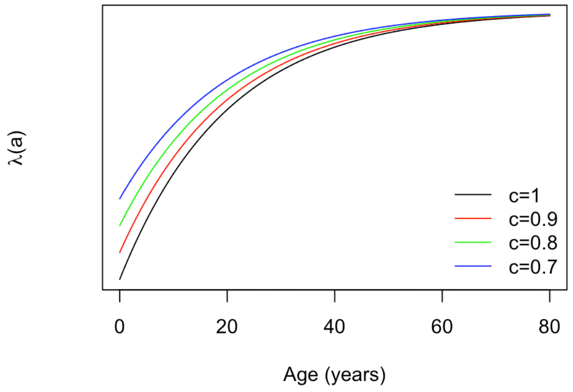

All three are free parameters being fitted to the malaria data, in addition to the rate of acquisition of clinical immunity (α), and one parameter to determine the extent of heterogeneity in risk given any fixed size for the HR group (unless homogeneity of assumed). This results in the estimation of 5 parameters in total (4 in the homogenous scenario).

3- The model predicts that "As much as 25 past malaria attacks are required in order to reduce by half the risk of a clinical malaria attack". Is this reasonable? Any other studies show this? It would be nice to see a discussion on this point.

Yes, we agree. We conceive the twenty-five malaria episodes encompassing both the number of infections (including overlapping superinfections) and relapses an average child experiences during his/her first years of life in holoendemic settings, e.g. in Africa, before effective clinical immunity is acquired. We thus argue that HR individuals in low-endemicity settings such as Latin America actually experience nearly as many repeated malaria episodes as the average child in rural Africa. We added to the Discussion some comments on this topic.

As I mentioned above, this is a good work, and deserves to be published in a good journal. But it would be interesting to have a more thorough discussion on the model assumptions and parameter "proliferation".

We thank the reviewer for the compliments. The basic assumptions for the model used to fit data are described in a section titled "The mathematical model" within Materials and Methods:

- "We assume an age-dependent force of infection λ(a) (Equation 1), which correlates mosquito biting activity with human body mass [23, 24]."
- "Also, assuming that individuals acquire partial immunity after repeated clinical malaria attacks, due to antibody- and cell-mediated responses [25], we introduce a factor describing the development of partial immunity. The strictly positive decreasing function σ(i) of the number i (i≥0) of past clinical vivax malaria attacks each individual has experienced (Equation S2)."
- "Assuming equilibrium with respect to time,…"

The model was further refined as described in section titled "Mathematical model with asymptomatic infections", also within Materials and Methods, for additional explorative analyses:

- "We refined the model with compartments comprising infected but asymptomatic individuals, by assuming that the proportion of asymptomatic infections depends on gradually acquired partial immunity."

- The final section “Asymptomatic parasite carriers, duration of infection and the infectious reservoir”, also within Materials and Methods, discusses several model scenarios considered for asymptomatic infections.
