# Supplementary material for: Modelling the epidemiology of residual Plasmodium vivax malaria in a heterogeneous host population: A case study in the Amazon Basin
Source: PLoS Comput Biol. 2020 Mar 13;16(3):e1007377. doi: 10.1371/journal.pcbi.1007377 (PMC7108741; doi:10.1371/journal.pcbi.1007377)
Supplement: Attachment — Submitted filename: Rebuttal_R2.pdf [file pcbi.1007377.s003.pdf]

PCOMPBIOL-D-19-01472

Modelling the epidemiology of residual *Plasmodium vivax* malaria in a heterogeneous host population: a case study in the Amazon Basin

Editor's comments:

Indeed, the model predicts that as much as 25 past clinical malaria attacks are required in order to reduce by half the risk of a clinical malaria attack. In holoendemic settings, children are typically continuously infected during the transmission season, with frequent superinfection and overlapping clinical malaria episodes during their first years of life.

Could the authors provide a reference? This is a strong and quantitative (25 cases) statement and it just floats around.

> This result is inferred from the model and its assumptions. Although it is not possible to find a reference to direct corroborate this result, we added two new references that, indirectly, dialog with it: one describing vivax malaria incidence rates in children in Papua New Guinea and another describing falciparum malaria incidence in children in Mali (lines 281-286). Both estimates give an average of 25 malaria attacks by age 10-11 years. Indeed, in such areas, malaria remains common throughout most of childhood, while a significant decrease in risk of infection is seen in adolescence and early adulthood. Whether this decrease in risk corroborates the factor (one half) suggested by our model is a hypothesis that can be tested by dedicated experimental or observational studies.
>
> We also add “on average” as follows:
>
> Indeed, the model predicts that as much as 25 past clinical malaria attacks, on average, are required in order to reduce by half the risk of a clinical malaria attack.

A reference for "In other words, HR individuals in our Amazonian study population are nearly as exposed to malaria as the average child living in rural Africa." would also be welcome.

Please see lines 293-30. We added some further clarifications.

As an aside: moving all equations and mathematical details to S1 resulted in a poorer text. Equations speak by themselves and are much clearer than a description in words. Anyhow, this was done because the other reviewer ask it, so I will not suggest to go back to the previous version.

We share the same option as this reviewer while appreciating the concerns of the other. As a compromise we move the model construction back to the main text and left the technicalities of parameters estimation as supplement.
